# Type 2 diabetes mellitus related sarcopenia: a type of muscle loss distinct from sarcopenia and disuse muscle atrophy

**DOI:** 10.3389/fendo.2024.1375610

**Published:** 2024-05-24

**Authors:** Zhenchao Liu, Yunliang Guo, Chongwen Zheng

**Affiliations:** ^1^ Institute of Integrative Medicine, Qingdao University, Qingdao, Shandong, China; ^2^ Department of Neurology, The 2^nd^ Affiliated Hospital of Fujian University of Traditional Chinese Medicine, Fuzhou, Fujian, China

**Keywords:** sarcopenia, disuse muscle atrophy, type 2 diabetes mellitus, muscle fibers, muscle loss

## Abstract

Muscle loss is a significant health concern, particularly with the increasing trend of population aging, and sarcopenia has emerged as a common pathological process of muscle loss in the elderly. Currently, there has been significant progress in the research on sarcopenia, including in-depth analysis of the mechanisms underlying sarcopenia caused by aging and the development of corresponding diagnostic criteria, forming a relatively complete system. However, as research on sarcopenia progresses, the concept of secondary sarcopenia has also been proposed. Due to the incomplete understanding of muscle loss caused by chronic diseases, there are various limitations in epidemiological, basic, and clinical research. As a result, a comprehensive concept and diagnostic system have not yet been established, which greatly hinders the prevention and treatment of the disease. This review focuses on Type 2 Diabetes Mellitus (T2DM)-related sarcopenia, comparing its similarities and differences with sarcopenia and disuse muscle atrophy. The review show significant differences between the three muscle-related issues in terms of pathological changes, epidemiology and clinical manifestations, etiology, and preventive and therapeutic strategies. Unlike sarcopenia, T2DM-related sarcopenia is characterized by a reduction in type I fibers, and it differs from disuse muscle atrophy as well. The mechanism involving insulin resistance, inflammatory status, and oxidative stress remains unclear. Therefore, future research should further explore the etiology, disease progression, and prognosis of T2DM-related sarcopenia, and develop targeted diagnostic criteria and effective preventive and therapeutic strategies to better address the muscle-related issues faced by T2DM patients and improve their quality of life and overall health.

## 1 Introduction

Muscle loss is a widely recognized health issue, particularly with the increasing trend of population aging and the emergence of the concept of sarcopenia, as well as the growing depth of research in this field. Sarcopenia refers to the loss of muscle mass, muscle strength, and function due to rapid degeneration of muscle tissue and atrophy of muscle fibers. It is considered one of the common pathological processes of muscle loss in the elderly, but can also occur in other populations (1). On the other hand, disuse muscle atrophy refers to muscle wasting caused by prolonged muscle inactivity, often occurring in situations of insufficient physical activity, prolonged bed rest, or weightlessness (2). Furthermore, with further research, muscle-related issues caused by chronic debilitating diseases have also been widely reported, leading to the concept of secondary sarcopenia. Worth noting is the muscle loss following Type 2 Diabetes Mellitus (T2DM), which is defined as secondary sarcopenia and referred to as “T2DM-related sarcopenia” (3).

Although all three muscle-related issues involve the loss of muscle mass, they differ in terms of pathological mechanisms and clinical manifestations. There is more debate regarding the accuracy and appropriateness of the current naming conventions for muscle loss following chronic diseases. In this review, we aim to compare and discuss these three conditions, providing a comprehensive comparative analysis. Through this review, we hope to deepen our understanding of the relationships among sarcopenia, disuse muscle atrophy, and T2DM-related sarcopenia, and propose new considerations for the accurate recognition and definition of T2DM-related sarcopenia. This will contribute to further research and clinical practice, enabling better management of these cases of muscle mass loss and improving individuals’ quality of life and overall health.

## 2 Comparison of sarcopenia, disuse muscle atrophy, and T2DM-related sarcopenia

### 2.1 Epidemiology and clinical presentation comparison

In 2010, the European Working Group on Sarcopenia in Older People (EWGSOP) defined sarcopenia as a syndrome characterized by progressive and generalized loss of skeletal muscle mass and strength, with the risk of adverse outcomes, including limb disabilities, decreased quality of life, and even mortality (4). Initially considered a geriatric syndrome affecting individuals over 65 years old, further research has revealed that sarcopenia can also occur in younger age groups. The 2018 EWGSOP consensus acknowledged that sarcopenia is not limited to the elderly population (5). Studies have shown that the prevalence of sarcopenia increases with age, with variations observed among different age and gender groups. For example, in the 60-64 age group, the prevalence of sarcopenia is approximately 14.3% among men and 20.3% among women. In contrast, the prevalence significantly rises to 59.4% among individuals aged 75 and older for men, and 48.3% for women (6). Gender differences have also been identified, with a higher proportion of women exhibiting lower skeletal muscle mass index (SMI) compared to men. Approximately 45% of men and 59% of women have an SMI below 1 standard deviation, while 7% of men and 10% of women have an SMI below 2 standard deviations (7). It is worth noting that different diagnostic criteria can lead to variations in the prevalence of sarcopenia (4, 5, 8–12) (see **Table 1**). For instance, using the EWGSOP 2010 criteria, the prevalence ranges from 0.4% to 57.4%, whereas using the Asian Working Group for Sarcopenia 2014 (AWGS 2014) criteria, the prevalence ranges from 0.3% to 53.0% (13). These discrepancies highlight the influence of diagnostic criteria and study populations on the reported prevalence of sarcopenia. Clinically, sarcopenia manifests primarily as a decline in muscle mass and function, leading to symptoms such as weakened muscle strength, reduced physical performance, and difficulty in daily activities. Furthermore, sarcopenia is associated with various health issues, including osteoporosis, increased risk of falls, swallowing difficulties, sleep disorders, depression, and an elevated risk of mortality from chronic diseases such as lung disease, liver disease, cancer, cardiovascular disease, and kidney disease (14–18). Age is the primary risk factor for sarcopenia, with other potential risk factors including male gender, inadequate physical activity, smoking, high blood glucose, and poor sleep quality (19). Hormones such as testosterone, estrogen, growth hormone, ghrelin, and vitamin D play important roles in regulating muscle mass and function, highlighting the close relationship between sarcopenia and the endocrine system. Decreased testosterone and estrogen levels, as well as deficiency in growth hormone and vitamin D, can contribute to muscle mass reduction and increase the risk of muscle atrophy (20–25).

**Table 1:** Diagnostic criteria and key thresholds for sarcopenia.

Diagnostic Criteria	Skeletal Muscle Mass index	Grip Strength (Kg)	Gait Speed (m/s)
EWGSOP 2010 (4)	SMI (Kg/m^2^) DXA male<7.0, famle<6.0	male<27, famle<16	≤0.8 (4m)
EWGSOP 2018 (5)	SMI (Kg/m^2^) DXA male<7.0, famle<6.0	male<27, famle<16	≤0.8 (4m)
IWGS (8)	SMI (Kg/m^2^) DXA male<7.23, famle<5.67	–	<1.0
AWGS2014 (9)	SMI (Kg/m^2^) DXA male<7.0, famle<5.4 BIA male<7.0, famle<5.7	male<20, famle<15	<1.0 (6m)
AWGS2019 (10)	SMI (Kg/m^2^) DXA male<7.0, famle<5.4 BIA male<7.0, famle<5.7	male<20, famle<15	<1.0 (6m)
FNIH (11)	DXA Appendicular Skeletal Muscle Mass/BMI (Kg/BMI) male<0.789, famle<0.512	male<26, famle<16	≤0.8
SSCWD (12)	Average level -2 standard deviations of healthy individuals aged 20-30 from the same ethnicity.	–	<1.0 or <400m(6min)

EWGSOP, European Working Group on Sarcopenia in Older People; IWGS, International Working Group on Sarcopenia; AWGS, Asian Working Group for Sarcopenia; FNIH, Foundation for the National Institutes of Health; SSCWD, Society on Sarcopenia, Cachexia and Wasting Disorders; SMI, Skeletal Muscle Mass Index; BMI, Body Mass Index; DXA, Dual Energy X.

The prevalence of disuse muscle atrophy lacks relevant research, but it is currently believed that the primary risk factors are long-term lack of physical activity or being in a microgravity environment. In terms of clinical presentation, disuse muscle atrophy primarily involves a reduction in local or overall muscle mass, resulting in atrophy and decreased size of the affected area. This condition is accompanied by a decline in muscle strength, limited movement function, and increased difficulty in performing daily activities. Flexibility and coordination are also diminished (2, 26). Unlike sarcopenia, which is considered a disease primarily affecting the elderly population, disuse muscle atrophy can be seen as a physiological change. The decrease in muscle protein synthesis caused by disuse muscle atrophy leads to the reduction in muscle mass and strength. However, this can be restored through physical activity and maintaining an active lifestyle, especially in younger individuals (27, 28).

Currently, the diagnosis of T2DM-related sarcopenia primarily relies on sarcopenia diagnostic criteria. However, there are significant variations among these criteria, leading to differences in prevalence rates and hindering the assessment and prediction of sarcopenia progression in T2DM. For example, de Freitas et al. (29) conducted sarcopenia screening in T2DM patients using the EWGSOP 2010 and 2018 diagnostic criteria, resulting in prevalence rates of 16.9% and 7%, respectively. Fung et al. (30) assessed T2DM patients using the AWGS criteria and found that 58% of elderly T2DM patients had pre-sarcopenia and sarcopenia, with 24% having sarcopenia and 4% having severe sarcopenia. Currently, only liver disease has established relevant diagnostic criteria for secondary sarcopenia (31). Given that the pathological changes of T2DM-related sarcopenia differ from sarcopenia, and the onset age is not limited to elderly patients, as muscle loss can also occur in some younger patients, and most of them still have normal muscle strength, which is not easy to detect (32–34). Therefore, developing targeted diagnostic criteria is a future issue that must be addressed. The clinical presentation of T2DM-related sarcopenia is similar to sarcopenia, with patients experiencing wasting, low muscle strength, and functional decline. Additionally, it can have an impact on patients’ prognosis and increase the risk of mortality (32). Like sarcopenia, advanced age remains a risk factor for T2DM-related sarcopenia. Male gender, chronic hyperglycemia, and osteoporosis are also potential significant risk factors, but more evidence is needed to support these associations (35). Sarcopenia can significantly affect the endocrine metabolism of T2DM patients. Nakanishi et al. (36) conducted a study on 1030 T2DM patients and found a certain association between sarcopenia and blood glucose control. After adjusting for other confounding factors, T2DM patients with sarcopenia had significantly higher levels of glycated hemoglobin (HbA1c) compared to those without sarcopenia. He et al. (37) also found that T2DM patients with sarcopenia had poorer nutritional status, imbalanced nutrient intake, deteriorating glucose metabolism, declining kidney function, and a higher risk of osteoporosis compared to the non-sarcopenic diabetic control group. Overall, these results suggest that sarcopenia may have detrimental effects on the endocrine regulation of T2DM, highlighting the need for further research to delve deeper into the relationship between sarcopenia and T2DM.

In summary, T2DM-related sarcopenia shares clinical characteristics with sarcopenia and disuse muscle atrophy. It involves a decline in muscle mass, low muscle strength, and impaired function. However, unlike sarcopenia, T2DM-related sarcopenia can occur not only in the elderly population but also in younger individuals. In addition, complications such as diabetic neuropathy and diabetic foot can lead to reduced physical activity in patients, which can result in muscle atrophy (38) and it may occur concurrently with sarcopenia and disuse muscle atrophy. (See **Table 2**).

**Table 2:** Comparison of sarcopenia, disuse muscle atrophy, and T2DM-related sarcopenia.

	Sarcopenia	Disuse muscle atrophy	T2DM related sarcopenia
Primary Causes	Aging	Prolonged disuse	T2DM
Onset Age	Often >65 years	Any age group	Any age group
Muscle Mass	Decreased	Decreased	Decreased
Muscle Strength	Decreased	Decreased	Decreased
Muscle Fiber Area	Decreased	Decreased	Decreased
Muscle Fiber Changes	Predominantly type II fibers	Predominantly type I fibers	Predominantly type I fibers
Muscle Cell Count	Decreased	Unchanged	Decreased
Muscle Self-Recovery	Difficult	Possible	Difficult

### 2.2 Comparison of pathological changes: changes in different types of muscle fibers

Skeletal muscle is a vital component of the human body, consisting of diverse muscle fibers that serve various physiological functions. Understanding the distinct characteristics and roles of these muscle fibers is crucial in differentiating conditions such as sarcopenia, disuse muscle atrophy, and T2DM-related sarcopenia. There are two primary types of muscle fibers in skeletal muscle: slow-twitch fibers (type I fibers) and fast-twitch fibers (type II fibers). These fiber types can be further divided into type IIa fibers (MHC-IIa) and type IIx fibers (MHC-IIx), each with unique properties based on their myosin heavy chain (MHC) isoform expression and ATPase activity (39, 40). Fast-twitch fibers are characterized by larger diameter, abundant muscle proteins, and high glycogen content. They possess high glycolytic capacity and excel in explosive movements like sprinting and weightlifting. Slow-twitch fibers, on the other hand, have a smaller diameter, more mitochondria, and high oxidative capacity. They rely on oxidative metabolism and are suited for endurance activities and maintaining posture. Both fiber types are crucial for muscle health, function, and overall physical performance. Additionally, hybrid fibers with varying characteristics exist in skeletal muscles to adapt to different physiological demands (39, 41). Research studies have shown significant differences in the proportion and distribution of fast-twitch and slow-twitch fibers in different muscle tissues and motor units. For example, the cricopharyngeus muscle, involved in respiration and phonation, has a higher proportion of slow-twitch fibers in its inner layer and relatively faster muscle fibers in its outer layer (42). Similarly, studies conducted on animal models have found variations in the proportion of fast motor units containing fast-twitch fibers in different muscle groups (43). These findings highlight the adaptability of muscle fibers to meet specific functional demands. Slow-twitch fibers, with their high oxidative capacity, are well-suited for sustained energy production during low-intensity exercises, while fast-twitch fibers are more suitable for rapid, explosive movements. For instance, the fast motor units in the fourth lumbrical muscle are particularly well-suited for activities requiring rapid power output (42–44). Studies on pork have further supported these concepts, showing that muscle composition is related to glycogen and lactate content. Muscles with lower glycogen and higher lactate content predominantly consist of type IIa and IIb fibers, while muscles with higher glycogen and lower lactate content mainly consist of type I fibers (45). In summary, different types of muscle fibers have distinct roles in muscle metabolism, enabling muscle tissues to develop adaptive characteristics according to functional demands and effectively cope with various types of movements and activities.

Sarcopenia, characterized by muscle fiber atrophy, has been found to be associated with aging in both animal and human studies. Specifically, there is a notable shift towards type II fiber atrophy (46, 47). Patients with sarcopenia also exhibit atrophy of type II muscle fibers (48, 49). Lamboley et al. (50) investigated the impact of high-intensity intermittent exercise and revealed that it may decrease the sensitivity of the contractile apparatus in type I muscle fibers, thereby reducing energy production during intense exercise. Additionally, a slight decrease in the specific force of type II muscle fibers may contribute to a decline in maximal strength. These type II fibers rely on rapid metabolic pathways to support sustained high-intensity exercise and efficient performance. To further explore the effects of aging on muscle fibers, Pugh et al. (51) conducted a study on Indian rhesus monkeys. Their findings revealed that pure type I fibers did not experience significant atrophy during the aging process. However, fibers expressing isoforms of type II myosin exhibited a reduced cross-sectional area. Interestingly, young monkeys displayed higher mitochondrial activity staining intensity in type II fibers, while middle-aged and old monkeys exhibited lower mitochondrial activity staining intensity. These observations suggest a potential decline in mitochondrial activity in type II fibers with age. Understanding these age-related changes is crucial in the context of mitochondrial’s pivotal role in cellular energy production, generating the majority of cellular energy. These research findings suggest that mitochondrial activity in type II fibers may decline with age. The above study indicates that when aging leads to type II fiber atrophy, muscle strength may decrease, the ability for rapid energy supply in muscles may be affected, resulting in reduced endurance and decreased athletic performance, which is consistent with the characteristics of sarcopenia.

Disuse muscle atrophy occurs when muscles are not used for a long time, such as during extended bed rest or in microgravity environments. This leads to a decrease in muscle mass, changes in muscle fiber type and structure, as well as a decline in metabolism and function. It is also associated with increased oxidative stress and inflammation (2, 52). Studies have shown that all types of muscle fibers undergo atrophy in disuse muscle atrophy, but type I fibers are more significantly affected. This can cause a shift in fiber type from type I and type IIa to type IIx, without a decrease in the number of muscle cells (53–56). In other words, the size and quantity of muscle fibers may change, but the number of muscle cells remains the same. Research on Indian langur monkeys by Pugh et al. (51) indicates that type I muscle fibers have the highest mitochondrial activity, indicating greater oxidative capacity. Similarly, a study by Shen et al. (57) on pork found that muscles rich in type I fibers have higher mitochondrial content and lower glycogen and glucose levels, leading to more efficient adenosine triphosphate (ATP) synthesis and lower glycolytic capacity. Therefore, a decrease in type I muscle fibers can affect energy production and metabolism by reducing the number of fibers capable of oxidative metabolism. This reduction in oxidative capacity can result in inadequate energy production during prolonged or high-intensity continuous exercise, affecting exercise performance and endurance. Additionally, changes in metabolism can influence the body’s energy balance, nutrient utilization, and regulation of energy expenditure.

It is hypothesized that type I fibers are relatively easier to recover in cases of disuse muscle atrophy due to their higher vascular density and oxidative enzyme content (58). Type I fibers also have higher oxidative metabolism capacity and oxygen demand, making them more likely to recover from disuse muscle atrophy caused by lack of exercise. Increased activity can quickly enhance oxygen supply and metabolism. In contrast, fast-twitch fibers rely more on glycolytic metabolism with lower oxygen demand, making recovery relatively more challenging after disuse muscle atrophy. Observations by Sharma et al. (59) on 15 patients with severe lower limb injuries suggest that slow muscle fibers have a relatively easier regeneration process compared to fast muscle fibers. However, further research is needed to confirm these findings.

Sarcopenia has been recognized as a complication of T2DM, and the two conditions mutually influence each other, posing a serious threat to the health of T2DM patients (60). Currently, there is a lack of research on muscle fiber changes specifically in T2DM patients with muscle wasting. To ensure the credibility and comprehensiveness of this review, we conducted a systematic literature search to obtain relevant research articles from multiple academic databases, including PubMed, Scopus, Google Scholar, J-stage, and Web of Science. We formulated a series of screening criteria. Firstly, we limited the language of the articles to English to ensure a comprehensive understanding and analysis of the selected literature. Secondly, the research objectives had to be closely related to T2DM, involving muscle biopsies and pathological changes in patients. Finally, we excluded non-original research articles such as review articles, book chapters, and conference abstracts, as well as articles unrelated to the topic of this review. Additionally, we limited the study design to include only clinical research and human experimental studies.

Through the aforementioned screening process, considering the inherent risks and difficulties for patients involved in muscle biopsies, as well as the requirement for informed consent, ethical review, and data protection, these factors may have limited the number and scope of relevant studies. Therefore, we only retrieved 8 muscle biopsy studies concerning changes in muscle fiber types in T2DM patients.

Through these studies, we found significant differences in pathological changes in sarcopenia with T2DM compared to sarcopenia and disuse muscle atrophy (See **Table 3**). In T2DM patients, unlike sarcopenia patients who primarily experience atrophy of type II muscle fibers, the changes in muscle fibers in T2DM patients are characterized by a reduction in type I fibers (61–63, 65, 68). Mårin et al. (61) observed a decrease in oxidative (type I) fibers and an increase in glycolytic (type II) fibers in non-insulin-dependent diabetes mellitus (NIDDM/T2MD) patients, particularly type IIB fibers. Hickey et al. (62) noted that the percentage of type I fibers was higher in the non-obese control group than in the obese NIDDM group, while the percentage of type IIb fibers was lower in the non-obese group compared to the obese NIDDM group. The proportion of type I fibers in the obese NIDDM group was also lower than in the obese group. Gaster et al. (63) found that T2DM patients had a higher average diameter of fast muscle fibers than slow muscle fibers, a significant decrease in the proportion of slow muscle fibers, and lower GLUT4 density in slow muscle fibers compared to fast muscle fibers. Oberbach et al. (65) reported a significant decrease in slow-twitch muscle fibers, an increase in fast-twitch muscle fibers, and reduced oxidative enzyme activity in muscles of T2DM patients compared to the healthy control group. Albers et al. (68) discovered that T2DM patients had a relatively lower proportion of type I muscle fibers and a relatively higher proportion of type IIx muscle fibers, suggesting that insulin has complex effects on muscle metabolism and signal transduction by regulating protein levels and phosphorylation states in muscle fibers. Although He et al. (64) did not observe significant differences in muscle fiber type compared to the healthy lean and obese control groups, their study still showed a downward trend in the proportion of type I fibers in T2DM patients, and they also found that the muscle oxidative enzyme activity in the T2DM group was significantly lower than that in the healthy lean group, while the lipid content was significantly higher. Additionally, Gaster et al. (63) found a significantly reduced density of glucose transporter 4 (GLUT4) in slow-twitch fibers of T2DM patients compared to the healthy control group. This may indicate a limitation in the muscle cells’ ability to uptake glucose, possibly due to disruptions in the insulin signaling pathway. Insulin typically stimulates the translocation of GLUT4 from intracellular vesicles to the cell membrane, increasing glucose uptake. Regarding the observation of type II fibers, Mogensen et al. (66) found a significantly higher number of type IIx fibers in T2DM patients compared to the obese control group, and the proportion of type IIx fibers was influenced by insulin resistance. Additionally, the study also found correlations between mitochondrial respiratory activity and metabolic indicators such as blood glucose control. Similar results were also observed in the study by Albers et al. (68), they found that compared to the healthy control group, T2DM patients have a relatively lower proportion of type I muscle fibers and a relatively higher proportion of type IIx muscle fibers. The study suggests that insulin plays a complex role in muscle metabolism and signal transduction by regulating protein levels and phosphorylation status in muscle fibers. And a study by Nagatomo et al. (69) in an animal model showed a significantly higher proportion of type IIx fibers in T2DM rats compared to the normal control group. Currently, the exact reasons for these changes are not fully understood, but they may represent a compensatory mechanism of the body. Type IIx fibers have a higher contraction speed and force-generating capacity, and in T2DM, where there is insulin resistance or insufficient insulin secretion, muscle cells may compensate for the inadequate glucose uptake and utilization by increasing the number of type IIx fibers. This compensatory mechanism may help diabetic patients maintain their ability to perform rapid and forceful muscle contractions. The aforementioned results suggest that changes in muscle fibers in T2DM patients are primarily influenced by the disease itself, and muscle damage in T2DM is influenced by the underlying mechanisms of the disease. However, these studies have limitations due to small sample sizes, and in the study conducted by Leenders et al. (67) with a relatively larger sample size, they found that compared to the healthy control group, T2DM patients showed no significant difference in the cross-sectional area of type I and type II muscle fibers, no significant difference in the proportion of muscle fibers between the two groups, and a significant correlation between the cross-sectional area of type II muscle fibers and lean body weight, skeletal muscle mass, leg extension, and leg push strength. Furthermore, there is currently a lack of research specifically focusing on T2DM sarcopenia patients. Choe et al. (70) research founded that categorizing pork into high, medium, and low blood glucose level groups revealed that compared to the medium and low blood glucose level groups, the high blood glucose group consisted of a lower proportion of type I fibers (6.44 vs. 8.77 *vs*. 8.56%, *P* < 0.05) and a higher proportion of type IIb fibers (85.68 *vs*. 82.09 *vs*. 82.24%, *P* < 0.05). The situation was reversed for the medium and low groups. Pearson correlation analysis showed a positive correlation between blood glucose levels and myofibrillar protein solubility (*r* ^2 =^ 0.193, *P* < 0.01), as well as a positive correlation with myosin heavy chain protein solubility (*r* ^2 =^ 0.342, *P* < 0.001), indicating a relationship between blood glucose levels and muscle fiber composition. Observation of adult pig muscles by Choe et al. (71) also found that groups with high blood glucose levels had a lower percentage of type I fibers and a higher percentage of type IIb fibers (3.95% *vs*. 88.15%), while the situation was reversed for the low blood glucose group (8.54% *vs*. 82.80%). The composition of muscle fiber types may affect blood glucose levels, subsequently impacting lactate and cortisol levels. Type I muscle fibers are primarily responsible for long-duration endurance activities and participate in aerobic metabolism. Patients with T2DM-related sarcopenia may have difficulty effectively utilizing oxygen and fatty acids to generate energy, leading to metabolic changes that affect energy metabolism and overall health.

**Table 3:** Literature on muscle fiber changes in T2DM patients.

Reference	Number of Participants (n)	Age (years)	Muscle Fiber Changes
MÅrin.et al. (61)	14	51±4	Compared to the control group without diabetes but with a comparable degree of obesity, patients with non-insulin-dependent diabetes mellitus (NIDDM/T2MD) showed a decrease in oxidative (type I) fibers and an increase in glycolytic (type II) fibers, particularly type IIB fibers.
Hickey.et al. (62)	10	42.7±1.3	The percentage of type I fibers in the non-obese control group was significantly higher than that in the obese NIDDM group, while the percentage of type IIb fibers in the non-obese control group was significantly lower than that in the obese NIDDM group. Additionally, the proportion of type I fibers in the obese NIDDM group was significantly lower than that in the obese group.
Gaster.et al. (63)	8	53.8±1.0	Compared to the healthy control group, patients with T2DM had a higher average diameter of fast muscle fibers than slow muscle fibers, and the proportion of slow muscle fibers significantly decreased; the GLUT4 density in slow muscle fibers was significantly lower than in fast muscle fibers, with a decrease of 18% in GLUT4 density in T2DM patients compared to the healthy control group.
He.et al. (64)	20	55±2	Compared to the healthy lean and obese control groups, there were no differences in muscle fiber type between the T2DM patients. However, the muscle oxidative enzyme activity in the T2DM group was significantly lower compared to the healthy lean group, while the lipid content was increased.
Oberbach.et al. (65)	10	58.7±6.4	Compared to the healthy control group, T2DM patients had a significant decrease in slow-twitch muscle fibers, the fast-twitch muscle fibers increased and reduced oxidative enzyme activity in the muscles.
Mogensen.et al. (66)	10	55±2	Compared to the obese control group, T2DM patients had an increase in the number of type II_X_ muscle fibers, and the maximal respiration of mitochondria through the electron transport chain is significantly reduced in patients with type 2 diabetes mellitus. Additionally, the study also found correlations between mitochondrial respiratory activity and metabolic indicators such as blood glucose control.
Leenders.et al. (67)	60	71±1	Compared to the healthy control group, T2DM patients had no significant difference in cross-sectional area of type I and type II muscle fibers, no significant difference in the proportion of muscle fibers between the two groups, and a significant correlation between cross-sectional area of type II muscle fibers and lean body weight, skeletal muscle mass, leg extension, and leg push strength.
Albers.et al. (68)	11	55±2	Compared to the healthy control group, T2DM patients had a relatively lower proportion of type I muscle fibers and a relatively higher proportion of type IIx muscle fibers. Insulin exerts complex effects on muscle metabolism and signal transduction by regulating protein levels and phosphorylation states in muscle fibers.

In summary, muscle fiber changes in T2DM-related sarcopenia differ from those in sarcopenia and disuse muscle atrophy. T2DM-related sarcopenia is primarily characterized by a reduction in type I muscle fibers and potential increase in type II fibers, which is a complication of diabetes. On the other hand, sarcopenia is characterized by age-related chronic reduction in muscle mass and strength, predominantly affecting type II fibers. Disuse muscle atrophy occurs during prolonged inactivity and is characterized by decreased muscle mass, changes in type I fiber type and structure, as well as metabolic and functional decline. These differences in muscle fiber changes likely contribute to inconsistencies in energy metabolism and various muscle cell functions, and are associated with the pathological changes in T2DM. Further research is needed to investigate the impact of these differences on patients’ metabolism and immune response.

### 2.3 Comparison of the pathogenesis of sarcopenia and disuse muscle atrophy

**Figure 1:**
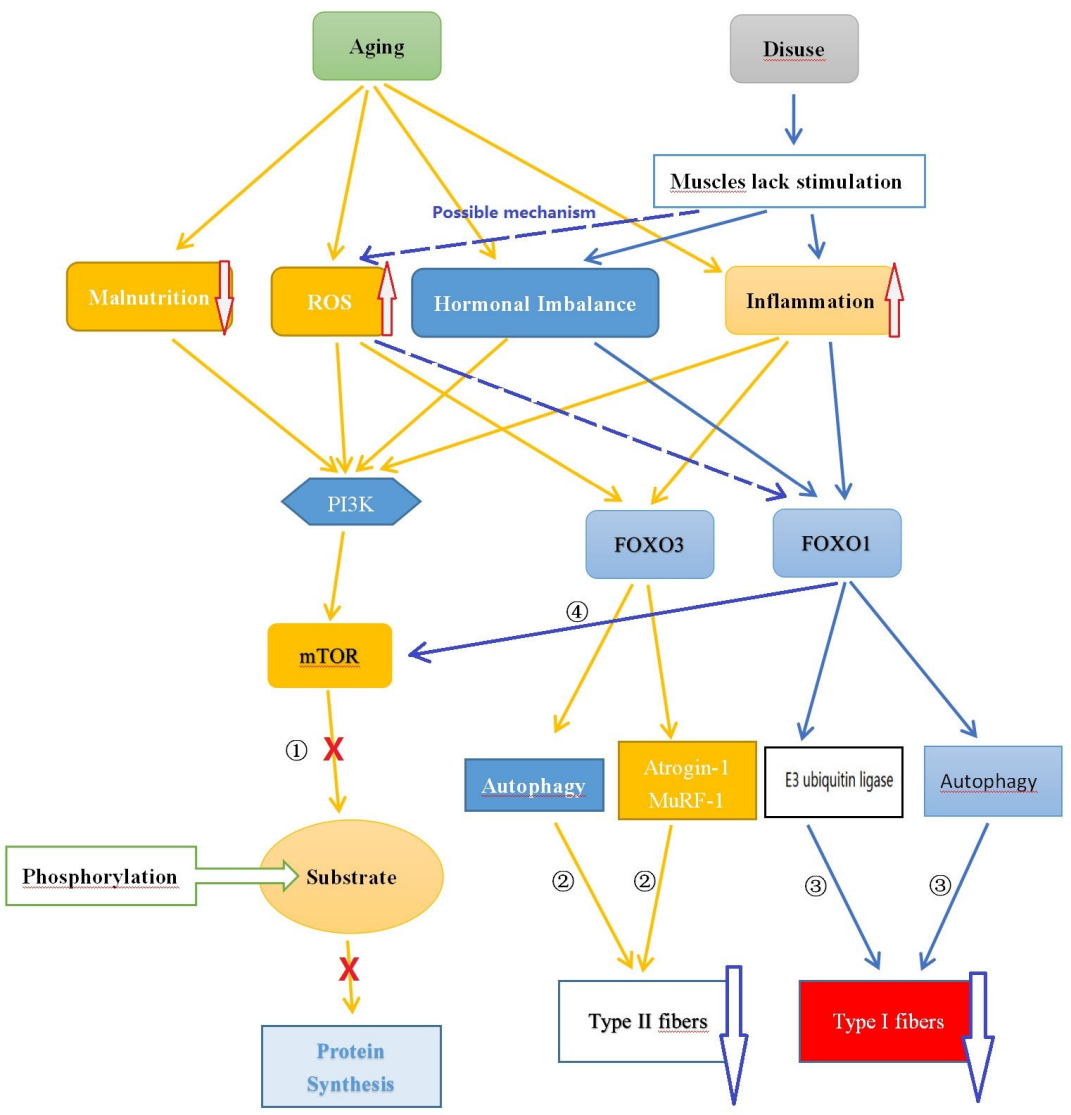
Comparison of the Potential Mechanisms of Sarcopenia and Disuse Muscle Atrophy. ① Aging leads to nutrient deficiency, chronic inflammation, endocrine imbalance, and oxidative stress, inhibiting mTOR pathway synthesis of muscle protein; ② Chronic inflammation and oxidative stress caused by aging promote FOXO3 leading to muscle protein degradation; ③ Reduced muscle stimulation caused by disuse results in hormone (such as testosterone) imbalance, chronic low-grade inflammation state causing muscle protein degradation through FOXO1; ④ FOXO1 can inhibit mTOR affecting muscle protein synthesis. ROS, Reactive Oxygen Species; FOXO, Forkhead Box O; PI3K, Phosphoinositide 3-kinase; AKT, Protein Kinase B; mTOR, Mammalian Target of Rapamycin.

Muscle protein synthesis is the process through which muscle cells produce new protein molecules to increase the size and quantity of muscle fibers. This process is regulated by multiple signaling pathways, including the mTOR (mammalian target of rapamycin) and Akt (protein kinase B, PKB) pathways. The mTOR pathway plays a critical role in promoting muscle protein synthesis by activating mTORC, which in turn phosphorylates various downstream substrates to enhance translation and peptide chain elongation, thereby increasing protein synthesis (72). The Akt pathway also contributes to the promotion of muscle protein synthesis. Akt controls protein synthesis and degradation by activating mTOR and regulating ribosome protein S6 kinase 1 (S6K1) and forkhead box O (FOXO). Akt phosphorylates FOXO transcription factors at multiple sites, leading to the exclusion of phosphorylated FOXO proteins from the cell nucleus and inhibiting their transcriptional function, thus suppressing the degradation of muscle proteins (73, 74). On the other hand, muscle protein degradation refers to the breakdown and degradation of protein molecules in muscle cells, a process in which the FOXO family of transcription factors plays a crucial role. All forms of FOXOs are expressed in skeletal muscle, with FOXO1 and FOXO3 being the most abundant. FOXO can activate the ubiquitin-proteasome and autophagy pathways. The ubiquitin-proteasome pathway is a major route involved in protein degradation, marking specific proteins as targets for degradation and degrading them through proteasomes, thereby facilitating protein degradation. Although autophagy plays a crucial role in maintaining cellular homeostasis and metabolic balance, and is involved in maintaining muscle homeostasis and function, an increase in autophagy-regulating proteins may help alleviate muscle apoptosis and oxidative damage, thereby maintaining the health of muscle tissue. However, autophagy decreases during normal aging, and this decrease is associated with an increase in abnormal mitochondria. These changes can lead to mitochondrial dysfunction and damage to muscle quality, ultimately leading to muscle atrophy. Furthermore, autophagy may also be excessively activated in sarcopenia, and excessive autophagy can result in muscle protein degradation and muscle loss (72, 75–77) (See **Figure 1**).

With aging, there is often a concurrent presence of malnutrition or inadequate nutrient intake, which can hinder muscle protein synthesis. Protein plays a crucial role in muscle synthesis, and a lack of protein intake can reduce the ability of muscle protein synthesis. Furthermore, other essential nutrients such as amino acids and vitamin D also have significant effects on muscle protein synthesis (78). Hormone levels also undergo changes as individuals age. For example, there is a decline in growth hormone (GH) and testosterone levels. These hormonal changes have the potential to affect muscle synthesis and maintenance (79–81). When GH binds to the receptors on the cell membrane, it activates the PI3K enzyme, which further phosphorylates PIP2 on the cell membrane, generating PIP3. Subsequently, PIP3 recruits Akt to the cell membrane and activates its phosphorylation process. The activated Akt inhibits the activity of FOXO, reduces protein degradation and autophagy, thus promoting muscle growth and maintenance. Additionally, Akt directly phosphorylates and activates mTOR, which enhances the signaling pathways related to protein synthesis and stimulates muscle cells to produce more protein, thereby promoting muscle growth (82). Testosterone can also impact the phosphorylation process of Akt/mTOR, thereby regulating their activity. Activated Akt suppresses the activity of FOXO (a transcription factor), reducing protein degradation and autophagy, thus promoting muscle growth and maintenance. Similarly, activated mTOR enhances the signaling pathways related to protein synthesis, prompting muscle cells to produce more protein and promoting muscle growth (83). These factors contribute to an imbalance between muscle protein synthesis and degradation, playing a crucial role in the development of sarcopenia.

Aging is characterized by the presence of chronic low-grade inflammation, characterized by increased concentrations of interleukin-6 (IL-6), tumor necrosis factor-α (TNF-α), and C-reactive protein (CRP) (84). These inflammatory factors can mediate mTOR and FOXO signaling through the ubiquitin-proteasome system and autophagy, leading to skeletal muscle protein loss. For example, excessive expression of IL-6 can induce cellular autophagy and muscle atrophy by activating JAK-STAT signaling pathways, while also promoting the upregulation of FOXO3a signaling, leading to activation of the ubiquitin-proteasome system and skeletal muscle protein degradation (85, 86). Excessive expression of TNF-α can activate the nuclear factor-κB (NF-κB) signaling pathway, which stimulates myostatin, a muscle growth inhibitor. Myostatin can inhibit skeletal protein synthesis through the Akt/mTOR pathway and regulate FOXO signaling, thus contributing to skeletal muscle atrophy (87, 88). As individuals age, cells produce more reactive oxygen species (ROS) in a resting state, leading to oxidative stress in the body (89). In a moderate range, ROS serves as a normal cell signaling molecule and plays an important role in regulating normal physiological processes. Appropriate levels of ROS can promote muscle differentiation and adaptive responses, thereby enhancing muscle strength and function. For example, Kim et al. (90) found that increased ROS levels can activate the PI3K/AKT/mTOR cascade, leading to mTORC1 activation through phosphorylation of unc-51-like autophagy-activating kinase 1 at the serine 317 site and upregulation of Atg protein expression, inducing autophagic signaling to further promote muscle differentiation, which is a critical step in muscle development and growth. However, high levels of ROS can inhibit protein synthesis by suppressing the PI3K/AKT/mTOR pathway and result in the overexpression of FOXO3, thereby triggering cellular autophagy and apoptosis. These mechanisms ultimately contribute to the development of muscle aging (91, 92).

**Figure 2:**
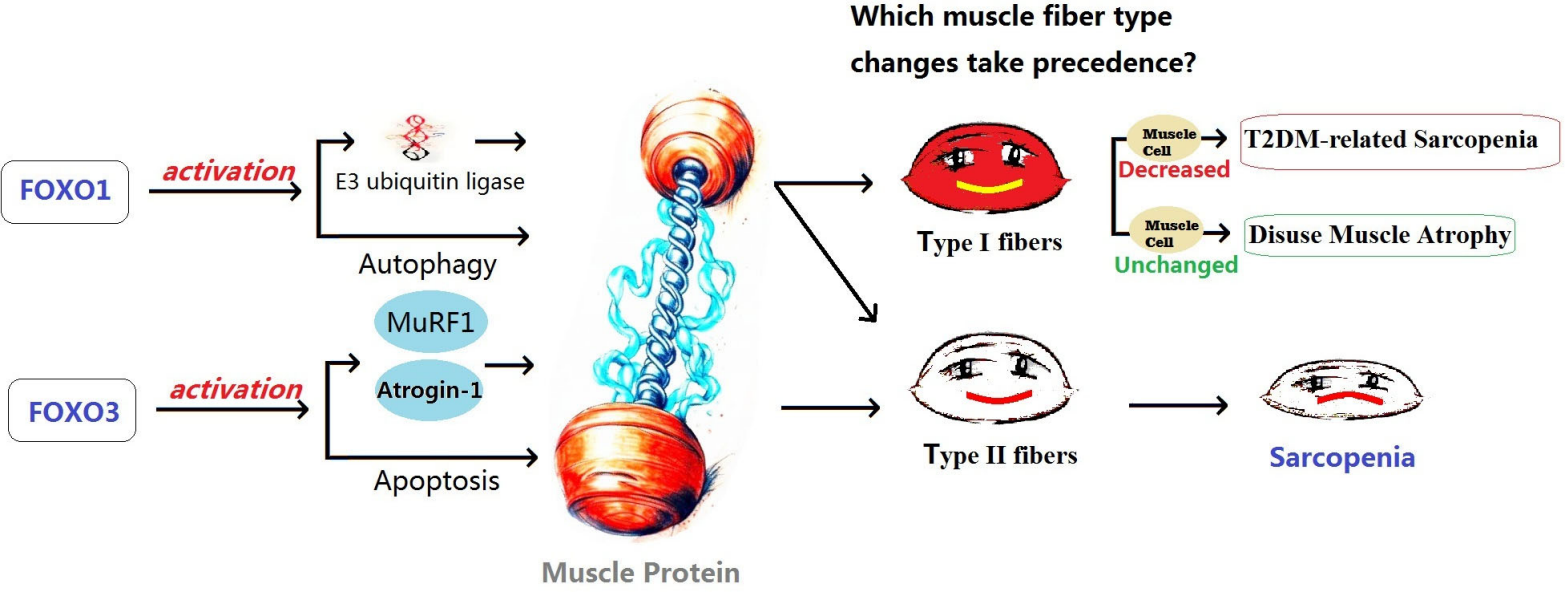
The possible effects of FOXO1 and FOXO3 on different types of muscle atrophy. T2DM, Type 2 Diabetes Mellitus; FOXO, Forkhead Box O.

Both FOXO1 and FOXO3 play a role in muscle atrophy, with FOXO1 potentially having a predominant role in disuse atrophy, while FOXO3 primarily mediates sarcopenia. High expression of FOXO3 has been detected in the muscles of sarcopenia patients. FOXO3 is believed to promote protein degradation by activating genes such as Atrogin-1 and MuRF1. It can also activate apoptosis-related genes, such as BNIP3, promoting cell apoptosis. Conversely, reducing FOXO3 expression can inhibit cell apoptosis, activate satellite cells, and promote their differentiation into muscle cells (93). Intervention studies targeting sarcopenia have shown that inhibiting the activity of FOXO3a and blocking the ubiquitin-proteasome signaling pathway mediated by FOXO3 can inhibit muscle protein degradation and promote muscle protein synthesis, providing therapeutic effects against sarcopenia (94, 95). On the other hand, the FOXO1 signaling pathway may primarily be involved in the development of skeletal muscle atrophy induced by disuse. FOXO1 is believed to be involved in muscle growth, metabolism, and cell differentiation, as well as regulating muscle fiber type specialization, with higher expression in muscles rich in fast-twitch fibers (96). FOXO1 plays an important role in regulating various proteases involved in protein degradation in muscles. It promotes protein degradation by activating E3 ubiquitin ligases such as MAFbx/atrogin-1 and MuRF1. It also affects muscle fibers by regulating cellular autophagy mechanisms. FOXO1 inhibits muscle cell differentiation by inhibiting the PI3K/Akt pathway. Additionally, it lowers the level of mTOR protein by reducing IGF-II expression, interfering with the differentiation process of myoblasts (96, 97). Studies on transgenic mice by Kamei et al. (98) showed that in skeletal muscles of FOXO1 mice, both the size of type I and type II fibers were significantly reduced, and the number of type I fibers was also significantly decreased. FOXO1 negatively regulates skeletal muscle mass and the expression of type I fiber genes, leading to impaired skeletal muscle function. Yuan et al. (99) also found that FOXO1 can enhance the expression of fast-twitch MyHC protein while inhibiting the expression of slow-twitch MyHC protein. Inhibition of FOXO1 may contribute to the transformation of fast fibers to slow fibers in the body. Studies by Vilchinskaya et al. (97) on a microgravity functional unloading rat model showed that FOXO1 can induce the transition of slow fibers to fast fibers through up-regulation of MuRF-1 and MuRF-2 expression, ultimately leading to disuse muscle atrophy. Although there have been studies suggesting that abnormal inflammatory factors and oxidative stress may play an important role in the muscle fiber changes induced by FOXO1, prolonged inactivity leads to the activation of FOXO1, which in turn activates a series of genes, including some related to inflammatory responses. The activation of these genes results in a sustained increase in inflammatory responses, leading to the occurrence of chronic low-grade inflammation, and leads to an increase in the production of ROS, an important signaling molecule responsible for mediating downstream muscle dysfunction and atrophy (99–102). However, there is still a lack of sufficient research to support the notion that long-term disuse can lead to abnormal inflammatory factors and increased ROS. Therefore, further investigation and more data are needed to support the understanding of the mechanisms underlying disuse muscle atrophy (See **Figure 2**).

### 2.4 Possible pathogenic mechanisms of T2DM-related sarcopenia

**Figure 3:**
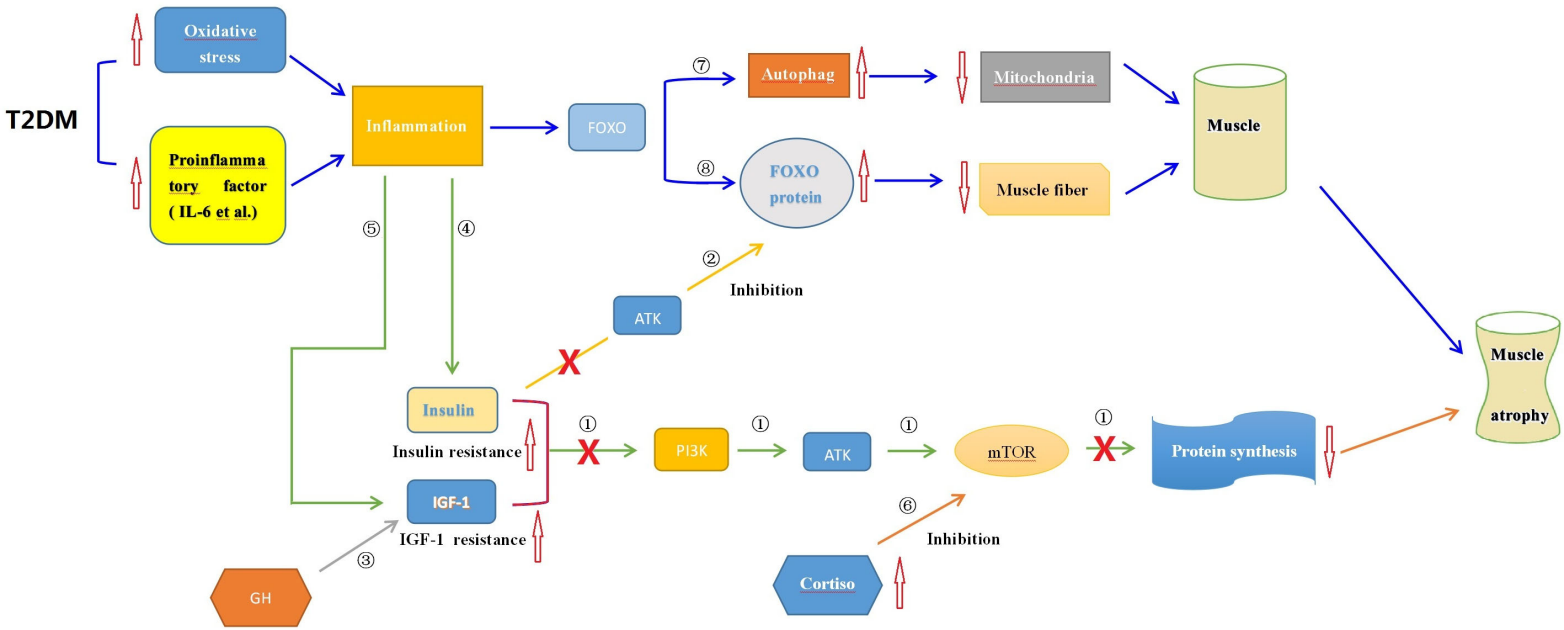
The possible endocrine mechanisms of muscle wasting associated with T2DM. ① Insulin, IGF-1, and other factors can activate the mTOR signaling pathway to promote muscle protein synthesis, thereby enhancing muscle growth and repair; ② Insulin and GH can inhibit the impact of FOXO on muscle protein degradation; ③ GH activates downstream mTOR through the Akt signaling pathway mediated by IGF-I, promoting protein synthesis; ④ Inflammatory conditions in T2DM induce insulin resistance, inhibiting protein synthesis as in mechanism①; ⑤ Inflammatory conditions in T2DM induce IGF-1 resistance, inhibiting protein synthesis as in mechanism①; ⑥Elevated levels of cortisol in T2DM suppress muscle protein synthesis by inhibiting the mTOR signaling pathway; ⑦ Inflammation triggers cell autophagy via the FOXO pathway leading to muscle protein degradation; ⑧ Inflammation activates FOXO proteins leading to muscle protein degradation. T2DM, Type 2 Diabetes Mellitus; mTOR, Mammalian Target of Rapamycin; FOXO, Forkhead box O; Akt, Protein Kinase B; PI3K, Phosphoinositide 3-kinase; IGF-I, Insulin-like Growth Factor-I; GH, Insulin and Growth Hormone; IL-6, Interleukin-6.

T2DM patients, similar to those with sarcopenia, exhibit chronic inflammation and increased oxidative stress, resulting in elevated levels of IL-6, TNF-α, and CRP. The loss of muscle in T2DM may be influenced by the PI3K/Akt/mTOR pathway, which regulates protein metabolism. However, the specific mechanisms by which these pathways affect muscle fiber, muscle cell metabolism, and neural fiber innervation in T2DM patients are still unclear and require further research (103, 104). Insulin resistance is another mechanism underlying T2DM-related sarcopenia. It is considered a major cause of muscle atrophy and is closely associated with the activity of mTORC and the FOXO family. The mTORC signaling pathway plays a significant role in regulating insulin sensitivity in skeletal muscle. Insulin activates mTORC1, promoting skeletal muscle protein synthesis. Inhibition of the mTORC1 pathway during muscle atrophy reduces muscle protein synthesis, leading to muscle loss. Insulin resistance can also induce skeletal muscle protein degradation through cellular autophagy (105–107). Insulin indirectly inhibits the activity of FOXO through Akt. Insulin resistance increases the expression of FOXO1, which inhibits mTORC1 activity. These factors contribute to abnormal muscle differentiation and muscle atrophy (108, 109). The changes in type I muscle fibers in T2DM patients may also be associated with insulin resistance. T2DM is a chronic endocrine disorder, and the changes in its muscle tissue are more complex than sarcopenia and disuse muscle atrophy. Elevated cortisol levels in T2DM patients may influence mTOR expression through different pathways, leading to muscle breakdown (110–113). Prostaglandins (PGs) have a dual effect on regulating insulin secretion and pancreatic β-cell proliferation, which are closely related to insulin resistance. They also play a significant role in muscle regulation. Upregulation of PGs may be associated with ferroptosis and iron metabolism disorders, leading to metabolic abnormalities and functional impairments in muscles (114, 115). PGs may also influence changes in muscle fiber types in T2DM patients and play a role in muscle growth and development (116, 117). Insulin-like growth factor (IGF-1) and growth hormone (GH) have been studied in relation to muscle mass and sarcopenia. IGF-1 promotes muscle growth and development, while GH activates mTOR and suppresses protein breakdown, ultimately increasing muscle mass (24, 118–120). The interaction between IL-6 and IGF-1 pathways can modulate inflammation response and alleviate sarcopenia (121). In summary, the occurrence of sarcopenia in T2DM patients is influenced by multiple factors, including cortisol, PGs, IGF-1, GH, and inflammatory factors. These factors collectively impact changes in muscle fiber types (See **Figure 3**).

In summary, malnutrition, hormonal changes, inflammatory state, and oxidative stress play important roles in muscle atrophy. The mechanisms involve multiple signaling pathways, including mTOR, Akt, and FOXO. However, the specific mechanisms are still not fully understood, and further research is needed to elucidate the precise mechanisms of mTOR, Akt, FOXO, and their interactions in different types of muscle atrophy. Additionally, in the case of T2DM patients, it is necessary to understand the distinct mechanisms underlying muscle alterations compared to sarcopenia, which can provide insights into the pathogenesis of T2DM itself. Further exploration of these mechanisms can enhance our understanding of muscle atrophy in T2DM patients and provide new strategies for prevention and treatment.

### 2.5 Exploring the endocrine significance of sarcopenia in T2DM

In the previous section, we discussed the possible mechanisms of sarcopenia in patients with T2DM. Among these mechanisms, the endocrine system plays a crucial role in regulating muscle growth and metabolism, including the influence of insulin, growth hormone, and testosterone on muscle. Therefore, understanding the complex interactions between the endocrine system and muscle metabolism is essential for preventing and treating muscle wasting associated with type 2 diabetes.

Sarcopenia in patients with T2DM can have various effects on glucose metabolism and energy utilization. Firstly, the atrophy of type I muscle fibers can lead to a decrease in energy metabolism efficiency. Type I muscle fibers are important for providing energy during endurance activities (122). A reduction in these fibers can result in insufficient energy supply, causing patients to feel more fatigued during long-duration or low-intensity endurance exercises. Secondly, the atrophy of type I muscle fibers may affect muscle stability and endurance. Type I fibers have slow contraction speed and fatigue resistance (123). Their decrease can reduce muscle endurance and stability, making patients more susceptible to muscle pain and fatigue. Additionally, the decrease in type I muscle fibers can impact glucose uptake and utilization. Type I fibers have high oxidative capacity and can convert glucose into energy (124). A reduction in these fibers can affect glucose uptake and utilization, leading to elevated blood glucose levels and worsening insulin resistance. Consequently, this can further exacerbate the patient’s glucose metabolism issues. In addition to the atrophy of type I fibers, T2DM patients also exhibit an increase in type II fibers. Soderlund et al. (125) studied muscle fiber types in T2DM patients compared to healthy volunteers and found differences in metabolic and contraction characteristics. Specifically, in T2DM patients, compared to healthy volunteers, type II fibers had higher resting levels of ATP, phosphocreatine, and glycogen. Additionally, the degradation rate of phosphocreatine and glycogen was higher in T2DM patients, especially in type IIb fibers, which may make T2DM patients more susceptible to fatigue during intense exercise.

Insulin resistance associated with T2DM can act on pathways such as mTOR and FOXO to promote muscle atrophy, leading to the occurrence of sarcopenia. Furthermore, there is a mutual promotion relationship between sarcopenia and insulin. Studies suggest that sarcopenia can affect lipid metabolism, leading to the accumulation of lipids and their derivatives inside and outside muscle cells. This lipid accumulation may interfere with normal metabolic activities within the cells, leading to mitochondrial dysfunction, disruption of fatty acid β-oxidation, enhanced production of reactive oxygen species, and subsequently causing insulin resistance, lipotoxicity, and increased secretion of certain inflammatory factors. These factors may lead to inflammation, ultimately establishing a vicious cycle of local inflammation and insulin resistance, which further exacerbates the development of sarcopenia (126, 127).

Sarcopenia in T2DM patients is mainly characterized by type I fiber loss. Animal studies have shown that different fiber types can affect insulin sensitivity. Muscles rich in type I and IIA fibers have a higher ability to uptake glucose under insulin stimulation, while muscles rich in type IIB and IIX fibers have lower insulin sensitivity (128). Therefore, if type I fibers decrease or type IIB fibers increase, it may lead to a decrease in insulin sensitivity, further exacerbating insulin resistance. This observation further emphasizes the important role of muscle fiber type in regulating insulin sensitivity. Additionally, type I muscle fibers have abundant mitochondria, which are the energy production centers within cells. Disrupted energy metabolism, due to impaired mitochondrial function in muscles, can negatively affect insulin action and contribute to insulin resistance (129).

Apart from insulin, patients with T2DM may also experience abnormal secretion of other hormones, such as growth hormone, leptin, and cholecystokinin (CCK). Leptin levels are associated with β-cell secretion and have been found to be abnormal in patients with sarcopenia. Furthermore, significantly elevated CCK concentrations have been observed in patients with sarcopenia. These hormones play important roles in appetite control and regulation of insulin secretion, and their abnormal secretion may have an impact on blood glucose control (130–133). This aberrant secretion may be related to conditions associated with sarcopenia, such as inflammation and oxidative stress. However, further research is needed, particularly regarding the unique changes in muscle fiber types in T2DM with sarcopenia, the resulting effects, and whether these effects differ from those caused by sarcopenia alone. Addressing these questions will require additional investigation.

## 3 Prevention and treatment strategies

Currently, there is no definitive method for preventing and treating muscle wasting disorders. Exercise and nutritional supplementation are widely recognized as intervention measures (29, 134). Resistance training has been shown to effectively slow down the progression of muscle wasting disorders. It stimulates muscle protein accumulation, promotes skeletal muscle protein synthesis, and improves muscle quality, strength, balance, and endurance. The underlying mechanisms may involve the activation of the Akt/mTOR signaling system and the inhibition of FOXO/MuRF1 expression, which positively affect protein metabolism and inhibit muscle protein breakdown (135–137). However, these studies lack sufficient support from basic research and clinical practice. Studies on disuse muscle atrophy suggest that it has the potential for self-recovery, unlike muscle wasting disorders (138). This potential recovery may be related to individual conditions and the duration of disuse, but further research is needed. In diabetic patients, the reduction of type I muscle fibers may lead to decreased energy metabolism efficiency, changes in muscle contraction characteristics, restricted glucose uptake, and decreased exercise capacity. Unlike disuse muscle atrophy, which is a physiological change, diabetic-related muscle wasting is a pathological condition and relatively difficult to recover from. Accurately distinguishing between types of fiber atrophy can help assess patients’ exercise capacity and predict disease progression. Therefore, preventive measures need to consider these differences. While endurance training and protein intake are effective for many cases of muscle wasting disorders, they may not necessarily apply to all types of muscle atrophy. For disuse muscle atrophy, which may be related to factors such as prolonged bed rest and lack of activity, the focus of prevention should be on promoting active participation in rehabilitation exercises and restoring activity capacity. For T2DM patients with muscle wasting, in addition to considering appropriate resistance training and high-intensity interval training, increasing the intake of foods rich in antioxidants and branched-chain amino acids is important. Special attention should also be given to blood sugar control, targeted treatment, and nutritional regulation due to the specific endocrine and metabolic abnormalities associated with diabetes that affect muscle. Regular assessment of muscle mass and function, as well as monitoring muscle health, are essential measures, especially for screening young patients.

## 4 Current issues and future perspectives

This review offers a comprehensive comparison and summary of sarcopenia, disuse muscle atrophy, and T2DM-related sarcopenia, focusing on pathological changes, epidemiology, clinical presentation, etiology, and prevention and treatment strategies. The findings demonstrate that these three conditions share similarities and differences in terms of etiology, age characteristics, pathological changes, clinical presentation, and disease mechanisms. Understanding these differences is crucial for accurately defining muscle-related issues in T2DM and other chronic diseases. Despite the widespread attention to sarcopenia, numerous unresolved issues and gaps in understanding T2DM-related sarcopenia persist. For instance, why T2DM-related sarcopenia primarily affects type I muscle fibers and what are the specific mechanisms underlying its pathogenesis? How do the pathological changes in T2DM itself contribute to muscle loss? What about muscle wasting in younger T2DM patients? Therefore, as a distinct form of muscle loss, T2DM-related sarcopenia requires further research to better understand its epidemiology, pathological mechanisms, and optimal diagnostic and treatment strategies. It cannot simply rely on the prevention and treatment strategies used for sarcopenia. More research is needed to address these questions. In future studies on muscle atrophy, it is essential to consider changes in muscle mass and function in different age groups and disease populations and to develop targeted interventions to improve individuals’ quality of life and health.

For future research, it is recommended to focus on the following aspects: first, conducting in-depth investigation of the pathological mechanisms and regulation of relevant signaling pathways in sarcopenia, disuse muscle atrophy, and secondary sarcopenia. Understanding these mechanisms is crucial for developing more effective prevention and treatment strategies. Second, further exploration of the role of exercise and nutritional interventions in muscle atrophy, including comparisons of different exercise modalities and the impact of nutritional supplementation on muscle mass and function. Large-scale epidemiological surveys and clinical data collection are needed to develop diagnostic criteria specific to T2DM-related sarcopenia. In conclusion, further research will contribute to a better understanding of the pathological mechanisms and prevention and treatment strategies for sarcopenia, disuse muscle atrophy, and T2DM-related sarcopenia, ultimately providing guidance to improve muscle mass and quality of life in individuals affected by these conditions.

## Author contributions

ZL: Writing – original draft, Writing – review & editing. YG: Writing – original draft. CZ: Writing – review & editing.
